# Non-Hodgkin’s Lymphoma presenting as deep venous thrombosis; A case report with literature review

**DOI:** 10.1016/j.ijscr.2020.05.006

**Published:** 2020-05-22

**Authors:** Okba F. Ahmed, Fahmi H. Kakamad, Abdulwahid M. Salih, Mohammed Q. Mustafa, Shvan H. Mohammed

**Affiliations:** a Mosul Cardiac Center, Mousl, Iraq; b Kscien Organization, Hamdi Str, Azadi Mall, Sulaimani, Kurdistan, Iraq; c Smart Health Tower, Madam Mitterrand Str. 46001, Sulaimani, Kurdistan, Iraq; d College of Medicine, Department of Surgery, University of Sulaimani, Sulaimani, Kurdistan, Iraq; e Department of Medical Analysis, Tishk International University, Erbil, Kurdistan, Iraq

**Keywords:** Deep venous thrombosis, Lymphoma, Lymphadenopathy

## Abstract

**Introduction:**

Idiopathic deep venous thrombosis is a relatively common clinical finding. The aim of this article is to report and discuss a case of lymphoma presented as DVT in line with SCARE guidelines with brief literature review.

**Case report:**

A 19-year-old male presented with right lower limb swelling for two week duration. Examination showed pitting leg edema with warm tense calf. Hematological investigations showed lymphopaenia. Duplex ultrasound showed DVT of right common femoral vein with inguinal lymphadenopathy. Computed tomography showed inguinal lymphadenopahthy. Histopathological examination of lymph node specimen confirmed the diagnosis of Non-Hodgkin’s Lymphoma (Burkett's type).

**Discussion:**

Several series have documented a significantly higher risk of malignancy in patients with presumed idiopathic DVT. Among such patients, 7.6% have been noted to have a malignancy during follow-up; the incidence of occult malignancy diagnosed within 6–12 months of an idiopathic DVT is 2.2–5.3 times higher than that expected from general population estimates.

**Conclusion:**

Idiopathic DVT regardless of age should be approached seriously with a special consideration to malignancy, lymphoma could present only with DVT.

## Highlights

• DVT presentation may be an atypical manifestation of an occult malignancy.
• Thorough search and work up for idiopathic DVT is mandatory.
• The aim of this article is to report a case of lymphoma presented as DVT with brief literature review.

## 1 Introduction

Unilateral lower extremity edema can be classiﬁed into acute or chronic case. The acute onset of unilateral leg edema points mostly to a diagnosis of thrombophlebitis, especially with the presence of inﬂammation such as hotness, tenderness and erythema [1]. A more insidious development or chronic pattern of unilateral leg edema can easily be misdiagnosed as venous insufﬁciency. In practice, such a clinical presentation could be an atypical appearance of an occult cancer leading to venous or lymphatic obstruction [1]. Deep venous thrombosis (DVT) pathogenesis is usually related to venous stasis (obstructive or non-obstructive), disruption of the vascular wall or hyper coagulopathy [2].

The aim of this article is to report and discuss a case of lymphoma presented as DVT in line with SCARE guidelines with brief literature review [3].

### 1.1 Patient information

A 19-year-old male presented to the outpatient clinic of vascular surgery complaining of gradually increased swelling involving his right lower limb (RLL) for two week duration. He was an accountant at a grocery shop with clear past medical and past surgical history.

### 1.2 Clinical findings

Clinical examination revealed a well-built man with body mass index of 25.8 kg/m^2^ with normal vital signs and not feverish. Examination of RLL showed pitting leg edema grade 2/4 with warm tense calf with 3.5 cm discrepancy with the other leg. Distal pulses were positive. Neurological examination was normal. Also there was discrete rubbery non-tender right inguinal lymphadenopathy of variable size of 1–4 cm.

### 1.3 Investigation

Hematological investigations showed lymphopaenia. Duplex ultrasound of RLL showed DVT of right common femoral vein with inguinal lymphadenopathy; largest one was 4 cm in size with an ill-defined mass 6 cm in size above the inguinal ligament encasing both external iliac artery & vein.

IV contrast enhanced CT-scan of abdomen & RLL showed enlarged lymph nodes encasing external iliac artery & common femoral artery. Excisional biopsy was planned & performed successfully under general anesthesia. Histopathological examination of the specimen confirmed the diagnosis of Non-Hodgkin’s Lymphoma (NHL) (Burkett’s type).

### 1.4 Therapeutic intervention

The patient was advised to have RLL bandaging, I.V anticoagulant and referred to hematological department for further management.

## 2 Discussion

Malignancy, surgery and trauma within the preceding three months remain the most significant risk factors for outpatient DVT, approximately 47% of outpatients with a documented DVT have one or more of the recognized risk factors, approximately 20% of all first-time acute venous thromboembolism i.e. DVT or pulmonary embolism (PE) events are associated with malignancy, an estimated 1 in 200 individuals with malignancy will develop either DVT or PE, a fourfold higher risk than those without malignancy [4].

Several series have documented a significantly higher risk of malignancy in patients with presumed idiopathic DVT. Among such patients, 7.6% have been noted to have a malignancy during follow-up; the incidence of occult malignancy diagnosed within 6–12 months of an idiopathic DVT is 2.2–5.3 times higher than that expected from general population estimates [4].

The most prevalent clinical presentation of NHL is inguinal or cervical lymphadenopathy. In many cases, patients notice masses in various part of the body and look for a physician’s advice. Generally, lymph nodes in case of lymphoma are non-tender, firm, and does not associated with a locao-regional infection. In other cases, lymphnode enlargement are found in more critical areas like the retroperitoneum or mediastinum leading to compression symtoms. Cough, chest pain, superior vena cava syndrome, abdominal pain, spinal cord compression, back pain and ureteral compression leading to symptoms of renal insufficiency are characteristics [5]. Elgendy and Margaret reported a 60-year-old Asian male presented with DVT of left lower limb, he had history of leg trauma and bedridden for few weeks, on examination he was revealed to have lymphoma. The current case was a young male with no history of trauma or immobilization [6].

Ronsdorf et al. searched 236 patients with proven DVT for occult cancer and found eleven cases. They concluded that the prevalence of occult malignancy in idiopathic DVT was higher than previously thought. They recommended that taking history, proper clinical examination, requesting simple laboratory tests with chest x.ray are the mandatory strategies that should never be forgotten when one deals with DVT. In their opinion, abdominal ultrasound adds nothing to the work up and can be omitted [7].

The investigators confirmed that vascular compression by the surrounding enlarged lymph nodes is the most significant risk factor for development of DVT in case of lymphoma [8]. The current case showed compression of the pelvic and upper thigh vessels by the multiple lymph nodes.

In addition, concerning the concept of Virchow’s triad which was named after the German pathologist, Rudolf Virchow, where he observed that thrombosis occurred when the following three situations were met; vascular wall (endothelial) injury, stasis of blood, and changes in the consistency of blood that enhances coagulation (hypercoagulability) with concerning stasis which occurs as a result of external compression of the vessel wall which eventually leads to thrombos formation as in the present case, other studies have supported this concept; Hisatomi et al. reported a case of DVT associated with iliac lymph node metastasis as a result of a primary unknown tumor [9,10]. Chiaramonte et al. reported a case iliac vein thrombosis as a result of endometriosis mass, Hassan et al. reported a case of iliac vein thrombosis as a result of impacted ureteric stone and Eltawansy et al. presented a case of jugular-subclavian vein thrombosis as a result of mediastinal lymphoma [11,12].

In this report, it will be clear that extensive search for underlying causes in case of DVT is of paramount to exclude a serious disease.

In conclusion, idiopathic DVT regardless of age should be approached seriously with a special consideration to malignancy, lymphoma could present only with DVT.

## Declaration of Competing Interest

There is no conflict to be declared.

## Funding

No source to be stated.

## Ethical approval

Approval is not necessary for case report in our locality.

## Consent

Consent has been taken from the patient and the family of the patient.

## Author contribution

Okba F. Ahmad: Surgeon managing the case and follow up, writing the manuscript.

Shvan H. Mohammed, Fahmi H. Kakamad, Berwn A.Abdulla and Diyar A. Omar: Writing the manuscript and follow up.

Abdulwahid M. Salih, Mohammed Q. Mustafa, Rawezh Q. Salih, Hunar Ali Hassan, Suhaib H.Kakamad, Hawbash M. Rahim, Jaafer O. Ahmed: literature review, final approval of the manuscript.

## Registration of research studies

Not applicable.

## Guarantor

Fahmi Hussein kakamad.

## Provenance and peer review

Not commissioned, externally peer-reviewed.
